# Clustering of lifestyle risk behaviours and its determinants among school-going adolescents in a middle-income country: a cross-sectional study

**DOI:** 10.1186/s12889-019-7516-4

**Published:** 2019-08-27

**Authors:** Chien Huey Teh, Ming Woey Teh, Kuang Hock Lim, Chee Cheong Kee, Mohd Ghazali Sumarni, Pei Pei Heng, Tajul Hassan Mohd Zahari, Ying Ying Chan, Md Iderus Nuur Hafiza, Eng Ong Tee, Kamaludin Fadzilah

**Affiliations:** 1 Institute for Medical Research, Ministry of Health, Jalan Setia Murni U13/52, 40170 Seksyen U13, Bandar Setia Alam, Malaysia; 2 Hospital Tengku Ampuan Rahimah, Jalan Langat, 41200 Klang, Selangor Malaysia; 3 Institute for Public Health, Ministry of Health, Jalan Bangsar, 50590 Kuala Lumpur, Malaysia; 4 Allied Health Science College, Jalan Hospital, 47000 Sungai Buloh, Selangor Malaysia

**Keywords:** Clustering, Multiple health risk behaviours, Risk factors, School-going adolescents, MyAHRB

## Abstract

**Background:**

Lifestyle risk behaviours such as smoking, alcohol consumption, physical inactivity, sedentary behaviour and low fruit/vegetable intake have been identified as the major causes of chronic diseases. Such behaviours are usually instigated in adolescence and tend to persist into adulthood. Studies on the clustering of lifestyle risk behaviours among adolescents are scarce, particularly in developing countries. Therefore, the present paper aimed to determine the clustering of lifestyle risk behaviours and its determinants among school-going adolescents in Malaysia.

**Methods:**

Data were extracted from a cross-sectional study, the Malaysian Adolescent Health Risk Behaviour (MyAHRB) study, which was conducted from May to September 2013 across 11 states in Peninsular Malaysia. A two-stage proportionate-to-size sampling method was employed to select a total of 3578 school-going adolescents aged 16–17 years from 20 selected schools in urban and rural settlements, respectively. The MyAHRB study adopted a set of self-administered questionnaires adapted from the Global School-based Student’s Health Survey (GSHS) and the Youth Risk Behaviour Surveillance.

**Results:**

The results from the analysis of 2991 school-going adolescents aged 16–17 years showed that 16 (in boys) and 15 (in girls) out of 32 combinations of lifestyle risk behaviours clustered. Girls (aOR 2.82, 95% CI: 2.32–3.43) were significantly more likely to have clustered risk behaviours than boys; however, no significant associated factors were observed among girls. In contrast, boys of Malay descent (aOR 0.64, 95% CI: 0.46–0.89) or boys who had at least three friends (aOR 0.65, 95% CI: 0.43–0.99) were less likely to engage in multiple risk behaviours.

**Conclusion:**

The present study demonstrated the clustering of multiple risk behaviours that occurred in both genders; these results suggest that multiple behaviour intervention programmes, instead of programmes based on siloed approaches, should be advocated and targeted to the high-risk sub-populations identified in the present study.

## Background

Adolescence is one of the critical transitions in human growth during which young adults undergo tremendous physiological and psychological developments to transition into adulthood [1]. Previous studies have demonstrated that adolescents tend to engage in a spectrum of lifestyle risk behaviours, such as smoking, drinking alcohol, being physically inactive, engaging in sedentary behaviour and having low fruit/vegetable intake [2, 3]; these unhealthy behaviours are more likely to continue into adulthood [4]. Therefore, due attention should be given to these preventable and modifiable lifestyle risk behaviours in view of their well-known contributions to the global burden of non-communicable diseases such as cardiovascular disease, some cancers, chronic respiratory disease and diabetes [5], particularly among adolescents.

In view of this, there have been extensive international and local studies on the magnitude and determinants of individual lifestyle risk behaviours among adolescents, such as smoking [6, 7], alcohol consumption [8, 9], physical inactivity [10, 11], sedentary behaviour [12, 13] and low fruit/vegetable intake [14, 15]. However, when these risk behaviours clustered more than expected by the independent occurrence of an individual risk behaviour, a clustered effect is observed [16]. Clustering lifestyle risk behaviours have a profound negative impact on overall health compared to the effect of individual risk behaviours since previous studies have demonstrated the synergistic rather than additive effects of risk behaviours on health, particularly on the increased risk for cardiovascular diseases [17, 18] and cancer [19, 20]. According to Jessor’s problem-behaviour theory, adolescents tend to be involved in more than one problem behaviour due to shared linkages of such behaviours in the social ecology of adolescence [21]. This clustering of risk behaviours is distinct from the co-occurrence of multiple risk behaviours, as the former occurs due to an underlying association between the co-occurring risk behaviours, while the latter merely describes the concurrent but independent engagement in multiple risk behaviours [22].

A number of studies have examined the prevalence of multiple co-occurring risk behaviours and their associated factors [3]. For instance, a study in San Diego in the United States reported that approximately 80% of adolescents aged 11 to 15 years had multiple risk behaviours (physical inactivity, sedentary behaviour, low fruit/vegetable intake, fat consumption) [23]. Dumith and colleagues also reported that 93.5% of Brazilian adolescents had co-occurrences of more than two risk behaviours (smoking, alcohol use, low fruit intake and/or physical inactivity), and adolescents who were female, were black or had a lower socioeconomic level were found to be more likely to have simultaneous occurrences of at least 3 risk behaviours than their corresponding counterparts [24].

Nonetheless, cross-sectional and cohort studies that demonstrate the magnitude and associated factors of clustered lifestyle risk behaviours among adolescents are scarce [3]. Previous observational studies that demonstrated the significant clustering effect of risk behaviours (smoking, alcohol consumption, physical inactivity, low fruit/vegetable intake) between men and women were conducted with adults [25, 26]. A systematic review conducted by Leech and colleagues in 2014 demonstrated that only eight studies have investigated the clustering of physical inactivity, sedentary behaviour and diet among children and adolescents, and these studies revealed that female, older age and lower socio-economic status were significant predictors for clustering of physical inactivity, sedentary behaviour and diet [2].

To the best of our knowledge, no study has attempted to explore the clustering of lifestyle risk behaviours and its associated factors among Malaysian adolescents. Therefore, the present paper aimed to determine the clustering pattern of five lifestyle risk behaviours (smoking, alcohol consumption, physical inactivity, sedentary behaviour and low fruit/vegetable intake) and investigate their associations with socio-demographic factors (gender, ethnicity, parental occupation, parental educational level, parental marital status, academic performance, and number of close friends) among school-going adolescents in Malaysia in order to identify high-risk sub-populations and hence could potentially guide the implementation of health promotion, education and interventional programmes pertaining to these lifestyle risk behaviours.

## Methods

### Study design

Data were extracted from the cross-sectional Malaysian Adolescent Health Risk Behaviour (MyAHRB) study, which was conducted from May to September 2013 across 11 states in Peninsular Malaysia. The sampling of schools was performed via a two-stage proportionate-to-size sampling method in order to ensure the representativeness of the findings. The first-stage stratification involved the selection of districts, and the second-stage stratification was performed by locality (urban/rural). An urban area was defined as a gazetted area with adjoining built-up areas of more than 10,000 people; otherwise, the area was categorised as a rural area. A total of 20 districts were selected from the 11 states, and subsequently, one secondary school per locality was selected from each of these districts via a simple random sampling method. All students who were 16–17 years old and had Malaysian citizenship from the 20 selected schools in urban areas and 20 selected schools in rural areas were recruited into the MyAHRB study. A design effect of 3 was used to adjust for the clustering effect within schools and districts. As a result, an optimum number of 3578 school-going adolescents aged 16–17 years were enrolled in the study. A detailed calculation of the optimum sample size of 3578 for the MyAHRB study was described elsewhere [7].

### Study instruments

The MyAHRB study employed a set of self-administered questionnaires adopted from the Global School-based Student’s Health Survey (GSHS) [27] and the Youth Risk Behaviour Surveillance [28] and adapted to the local socio-cultural context. The MyAHRB questionnaire was translated and back translated by a panel of language and content experts into our national language, Bahasa Malaysia, to establish content validity. The questionnaire was also pre-tested in selected schools in the state of Kuala Lumpur in a pilot study to establish face validity.

This validated self-administered questionnaire has two components: socio-demographics (age, gender, ethnicity, parental occupation, parental education level, parental marital status, family size, and self-perceived academic performance) and health behaviours (alcohol use, dietary behaviour, self-perceived body mass index (BMI) status, illicit drug use, mental health, physical inactivity, unsafe sexual behaviours, tobacco use, violence and unintentional injuries, and road safety).

### Data collection

Prior to data collection at the selected schools, written passive consent was obtained from the parents/guardians of the selected students with the assistance of the school administrators. Parents/guardians who did not allow their children to participate in the MyAHRB study were asked to return the consent forms, and these students did not participate in this study. During the data collection day, only students with passive consent were recruited into the study and briefed on the self-administered questionnaire by properly trained research team members. No teachers were allowed to be present during the questionnaire-answering session. All completed anonymous questionnaires were sealed in envelopes to ensure the confidentiality of the data.

### Smoking

Current smoking status was measured by a closed question, “In the last 30 days, on how many days have you smoked?”. Respondents who answered “1 to 2 days”, “3 to 5 days”, “6 to 9 days”, “10 to 19 days”, “20 to 29 days” or “every day” to the item were categorised as current smokers, whilst respondents who answered “no” were categorised as non-smokers.

### Alcohol consumption

Current drinking behaviour was assessed by a closed question, “In the last 30 days, on how many days have you had at least one alcoholic beverage?”. Respondents who answered “1 to 2 days”, “3 to 5 days”, “6 to 9 days”, “10 to 19 days”, “20 to 29 days” or “every day” to the item were categorised as current drinkers, whilst respondents who answered “no” were categorised as non-drinkers.

### Physical inactivity

Respondents who reported “0 days” to the item “During the past 7 days, on how many days were you physically active for a total of at least 60 minutes per day?” were considered inactive, and those who reported at least 1 day of engagement in at least 60 min of physical activity were considered physically active [29].

### Sedentary behaviour

Respondents who answered “3–4 h/day”, “5–6 h/day”, “7–8 h/day” or “more than 8 hours/day” to the item “How much time do you spend during a typical or usual day sitting and watching television, playing computer games, talking with friends, or doing other sitting activities?” were categorised as sedentary; whilst those answered “1–2 h/day” or “less than 1 hour/day” were classified as not being sedentary [30].

### Low fruit/vegetable intake

Two items were used to assess the level of fruit and vegetable intake, namely, “In the last 30 days, how many times per day have you eaten fruits?” and “In the last 30 days, how times per day have you eaten vegetables?”. In the present study, the number of times the respondents ate a fruit/vegetable was considered as the proxy for the number of servings of fruits/vegetables consumed. Therefore, respondents who had eaten fruits and/or vegetables at least 5 times per day were considered to have adequate intake of fruit and vegetables, whilst respondents who failed to do so were considered to be consuming an inadequate amount of fruit and vegetable [31].

### Data analysis

Descriptive analyses by gender were employed to depict the socio-demographic profile and individual prevalence of smoking, alcohol consumption, physical inactivity, sedentary behaviour and low fruit/vegetable intake as well as the total risk score of the recruited respondents. All five risk behaviours were dichotomised (presence of risk behaviour = 1; absence of risk behaviour = 0), and thus, each respondent was assigned a total risk score ranging from 0 to 5. For instance, respondents who had none of the five risk behaviours had a risk score of 0, while respondents with all five risk behaviours had a score of 5.

The clustering of lifestyle risk behaviours (smoking, alcohol consumption, physical inactivity, sedentary behaviour and low fruit/vegetable intake) were determined via the ratio of observed-to-expected prevalence (O/E). Clustering occurred when the observed prevalence (O) of a particular combination of lifestyle risk behaviours exceeded the expected prevalence of the combination (E) based on the random occurrence of the corresponding risk behaviours, which is when the O/E ratio is more than 1.0 [16].

Subsequently, in order to elucidate the associated factors for clustered risk behaviours (combinations of risk behaviours that had an O/E ratio of more than 1.0), multivariable binary logistic regressions were performed. The dependent variable, clustering of risk behaviours, was computed as follows: individuals with combinations of risk behaviours that had an O/E ratio of more than 1.0 (clustered risk behaviours) were coded as “1”, while those with combinations of risk behaviours that had an O/E ratio of less than 1.0 were coded as “0”. Independent variables such as ethnicity (Malay, non-Malay), parental education level (no formal education, primary school, secondary school, tertiary education), parents’ occupation (management and clerical, manual workers, not working), parents’ marital status (married, divorced), self-perceived academic performance (good, fair or poor) and number of close friends (fewer than three, at least three) were included simultaneously in the binary logistic regression model in order to obtain adjusted odd ratios (ORs). These analyses were performed by gender since there were significant interactions between gender, parental educational level and occupation. Hosmer–Lemeshow values of 0.701 (overall), 0.920 (for boys) and 0.739 (for girls) indicated that the data fit the binary logistic regression models well. All statistical analyses were performed at a 95% confidence interval using SPSS statistical software version 20 (IBM Corp., Armonk, NY, USA).

## Results

Of the 3578 eligible respondents, 83.6% (*n* = 2991) participated in the MyAHRB study. Among the participated respondents, 84.9% (*n* = 2538) of them provided a complete response to all five lifestyle risk behaviours of concern, whilst those with incomplete responses were excluded from the present study (*n* = 453). Further analyses of socio-demographic profiles between the study sample and those with incomplete information for the five lifestyle risk behaviours revealed nonsignificant differences (data not shown) and hence precluded non-response bias. As depicted in Table 1, approximately nine in ten adolescents were physically inactive (85.5%), seven in ten reported low intake of fruit/vegetable (70.4%), more than half were sedentary (56.0%), 14.6% smoked, and 5.0% reported alcohol consumption. For the total number of risk factors, 2.0% of the adolescents did not have any lifestyle risk behaviours, 14.9% had only one risk behaviour, and 0.6% had all five risk behaviours. More than one-third of the adolescents had simultaneous occurrence of 2 (39.7%) or 3 (37.1%) risk behaviours, while 5.8% had four risk behaviours that occurred simultaneously.

**Table 1:** Socio-demographics and lifestyle risk behaviour profile of 2538 school-going adolescents in Peninsular Malaysia, MyAHRB 2013

Variable	All		Boys		Girls	
	n	%	n	%	n	%
	2538		1214	47.8	1324	52.2
Ethnicity						
Malay	2014	79.4	943	77.7	1071	80.9
Non-Malay	524	20.6	271	22.3	253	19.1
Father’s Occupation						
Management and clerical	504	22.2	222	20.6	282	23.7
Manual workers	1670	73.7	807	74.9	863	72.5
Not working	93	4.1	48	4.5	45	3.8
Mother’s Occupation						
Management and clerical	501	21.6	212	19.5	289	23.5
Manual workers	294	12.7	143	13.2	151	12.3
Not working	1520	65.7	732	67.3	788	64.2
Father’s Education Level						
No formal education	52	2.1	22	1.9	30	2.3
Primary	215	8.7	84	7.1	131	10.2
Secondary	1464	59.4	717	61.0	747	58.0
Tertiary	733	29.7	352	30.0	381	29.6
Mother’s Education Level						
No formal education	55	2.2	31	2.6	24	1.9
Primary	224	9.1	102	8.7	122	9.4
Secondary	1609	65.0	780	66.2	829	64.0
Tertiary	586	23.7	266	22.6	320	24.7
Parents’ Marital Status						
Married	2377	94.6	1137	94.5	1240	94.6
Divorced	137	5.4	66	5.5	71	5.4
Academic Performance						
Poor/Fair	972	38.5	538	44.4	434	33.0
Good	1555	61.5	674	55.6	881	67.0
Number of Close Friend						
Fewer than 3	370	14.6	145	12.0	225	17.1
At least 3	2158	85.4	1064	88.0	1094	82.9
Lifestyle Risk Behaviour						
Smoking	371	14.6	337	27.8	34	2.6
Alcohol use	127	5.0	80	6.6	47	3.5
Physical inactivity	2171	85.5	983	81.0	1188	89.7
Sedentary behaviour	1422	56.0	656	54.0	766	57.9
Low food/vegetable intake	1787	70.4	850	70.0	937	70.8
Number of Risk Factor/Risk Score						
0	50	2.0	28	2.3	22	1.7
1	378	14.9	179	14.7	199	15.0
2	1007	39.7	434	35.7	573	43.3
3	941	37.1	442	36.4	499	37.7
4	147	5.8	122	10.0	25	1.9
5	15	0.6	9	0.7	6	0.5

As demonstrated in Table 2, the clustering effect, which is when the observed prevalence of a particular combination of risk behaviours was higher than the expected prevalence based on the random occurrence of the individual risk behaviours, was detected in 16 and 15 out of 32 different combinations of lifestyle risk behaviours in boys and girls, respectively. Generally, the O/E ratios were higher in girls than in boys. Among the girls, the clustering of all five risk behaviours was strongest across the 32 combinations with an O/E ratio of 13.53. The highest O/E ratio observed in boys was 4.38, where smoking, alcohol consumption and sedentary behaviour were clustered. It was notable that the clustering of smoking and alcohol use together with any of the other three risk behaviours was observed in both genders. Specifically, the clustering of smoking, alcohol consumption and sedentary behaviour (O/E ratio = 4.38), and smoking, alcohol consumption, sedentary behaviour and low fruit/vegetable intake (O/E ratio = 1.72) were strongest in boys. On the other hand, the clustering of smoking, alcohol consumption and physical inactivity (O/E ratio = 7.50), and smoking, alcohol consumption, physical inactivity and sedentary (O/E ratio = 10.92) were strongest in girls. In addition, it was noteworthy that the individual prevalence of low fruit/vegetable intake was less prevalent than the expected level (O/E ratio = 1.0 in boys and 0.68 in girls); however, when it co-occurred with smoking, alcohol use and physical inactivity, the observed prevalence was 38% greater in boys (O/E ratio = 1.38) and 210% greater in girls (O/E ratio = 3.10) than the expected prevalence that the four risk behaviours occurred randomly in the study population.

**Table 2:** Clustering of lifestyle risk behaviours among school-going adolescents in Peninsular Malaysia by gender, MyAHRB 2013

Number of risk factors	Smoking	Alcohol use	Physically inactive	Sedentary behaviour	Low fruit/vegetable intake	Boys			Girls		
						**O (%)**	**E (%)**	**O/E**	**O (%)**	**E (%)**	**O/E**
0	–	–	–	**–**	–	2.31	1.77	**1.30**	1.66	1.19	**1.40**
1	+	–	–	–	–	0.99	0.68	**1.45**	0.00	0.03	0.00
	–	+	–	–	–	0.16	0.12	**1.32**	0.15	0.04	**3.45**
	–	–	+	–	–	7.25	7.53	0.96	10.95	10.39	**1.05**
	–	–	–	+	–	2.22	2.08	**1.07**	1.96	1.63	**1.20**
	–	–	–	–	+	4.12	4.13	1.00	1.96	2.88	0.68
2	+	+	–	–	–	0.08	0.05	**1.72**	0.00	0.00	–
	+	–	+	–	–	3.21	2.89	**1.11**	0.23	0.27	0.83
	+	–	–	+	–	1.15	0.80	**1.44**	0.00	0.04	0.00
	+	–	–	–	+	1.57	1.59	0.99	0.00	0.08	0.00
	–	+	+	–	–	0.49	0.53	0.93	0.15	0.38	0.40
	–	+	–	+	–	0.16	0.15	**1.12**	0.15	0.06	**2.51**
	–	+	–	–	+	0.08	0.29	0.28	0.15	0.11	**1.43**
	–	–	+	+	–	7.33	8.85	0.83	12.99	14.26	0.91
	–	–	+	–	+	18.04	17.58	**1.03**	25.76	25.15	**1.02**
	–	–	–	+	+	3.62	4.86	0.75	3.85	3.95	0.97
3	+	+	+	–	–	0.08	0.20	0.40	0.08	0.01	**7.50**
	+	+	–	+	–	0.25	0.06	**4.38**	0.00	0.00	–
	+	+	–	–	+	0.00	0.11	0.00	0.00	0.00	–
	+	–	+	+	–	3.21	3.40	0.94	0.45	0.38	**1.21**
	+	–	+	–	+	6.01	6.76	0.89	0.38	0.66	0.57
	+	–	–	+	+	1.65	1.87	0.88	0.15	0.10	**1.45**
	–	+	+	+	–	0.82	0.62	**1.32**	0.30	0.52	0.58
	–	+	+	–	+	0.91	1.24	0.73	0.60	0.93	0.65
	–	–	+	+	+	23.15	20.67	**1.12**	35.50	34.52	**1.03**
	–	+	–	+	+	0.33	0.34	0.96	0.23	0.15	**1.56**
4	+	+	+	+	–	0.25	0.24	**1.03**	0.15	0.01	**10.92**
	–	+	+	+	+	1.24	1.46	0.85	1.06	1.27	0.83
	+	–	+	+	+	7.58	7.94	0.95	0.60	0.91	0.66
	+	+	–	+	+	0.33	0.13	**2.50**	0.00	0.00	–
	+	+	+	–	+	0.66	0.48	**1.38**	0.08	0.02	**3.10**
5	+	+	+	+	+	0.74	0.56	**1.32**	0.45	0.03	**13.53**

Values in boldface indicate presence of clustering effect

Girls (aOR 2.82, 95% CI: 2.32–3.43) were found to be significantly more likely to have clustered lifestyle health risk behaviours than boys after we controlled for socio-demographic factors (data not shown). However, no significant determinants of the clustering of risk behaviours were observed among girls (Table 3). On the other hand, boys who were of Malay descent (aOR 0.64, 95% CI: 0.46–0.89; non-Malay as the reference group) or who had at least three friends (aOR 0.65, 95% CI: 0.43–0.99; fewer than three friends as the reference group) were less likely to engage in clusters of lifestyle risk behaviours (Table 3).

**Table 3:** Adjusted odds ratios (aORs) of the clustering of health risk behaviours among school-going adolescents in Peninsular Malaysia, MyAHRB 2013

Variable	Boys			Girls		
	Adjusted OR	95% CI		Adjusted OR	95% CI	
		Lower	Upper		Lower	Upper
Ethnicity						
Malay	*0.64	**0.6**	**0.89**	0.66	0.432	1.00
Non-Malay	Ref			Ref		
Father’s Occupation						
Management and clerical	1.20	0.61	2.39	0.50	0.19	1.28
Manual workers	0.95	0.51	1.77	0.58	0.24	1.42
Not working	Ref			Ref		
Mother’s Occupation						
Management and clerical	1.06	0.72	1.58	1.05	0.67	1.63
Manual workers	0.98	0.64	1.49	0.91	0.56	1.47
Not working	Ref			Ref		
Father’s Education Level						
No formal education	Ref			Ref		
Primary	1.52	0.41	5.65	1.78	0.53	5.97
Secondary	2.14	0.62	7.43	1.32	0.43	4.20
Tertiary	2.09	0.58	7.48	1.36	0.40	4.62
Mother’s Education Level						
No formal education	Ref			Ref		
Primary	0.56	0.18	1.73	0.38	0.10	1.47
Secondary	0.60	0.21	1.75	0.74	0.20	2.78
Tertiary	0.56	0.18	1.74	0.61	0.15	2.49
Marital Status						
Married	1.05	0.56	1.97	1.18	0.58	2.40
Divorced	Ref			Ref		
Academic Performance						
Poor/Fair	Ref			Ref		
Good	1.03	0.79	1.4	0.85	0.61	1.17
Close friends						
Fewer than 3	Ref			Ref		
At least 3	*0.65	**0.43**	**0.99**	0.95	0.64	1.43

*Significant values at 95% confidence interval

## Discussion

This is the first study to investigate the clustering pattern of five major lifestyle risk behaviours (smoking, partake of alcohol, physical inactivity, sedentary behavior and inadequate fruit or vegetable intake) of chronic non-communicable diseases [32] and its determinants among school-going adolescents aged 16–17 years in the multi-ethnicity country of Malaysia with diverse socio-demographics and cultural backgrounds.

Generally, two major approaches are commonly used by researchers to analyse multiple risk behaviours, namely, analysis of co-occurrence of two or more risk behaviours and analysis of clustering of co-occurring risk behaviours. However, the analysis of co-occurrence, which merely examines the prevalence of different combinations of risk behaviours and/or the prevalence of the sum of risk scores, cannot demonstrate the underlying association between the co-occurring behaviours as the analysis of clustering can. The analysis of clustering, on the other hand, can be performed via two approaches: the first is via the computation of the observed prevalence-to-expected prevalence (O/E) ratio, and the second is via statistical methods such as factor analysis, latent class analysis and cluster analysis. Nonetheless, the cluster analyses are subjected to disputable issues about how clusters are defined and operationalised [22]. Since this study is the first of its kind in Malaysia, the O/E ratio method that is able to illustrate the detailed clustering effect of all possible combinations of the five risk behaviours of concern were chosen.

Previous studies have demonstrated the clustering of health risk behaviours among adolescents via the O/E ratio method. However, comparisons of findings between the present study and previous similar studies have to be performed with caution due to the variations in investigating different risk factors and defining terms and cut-off points for the dichotomisation of risk factors as well as differing age groups and targeted populations of recruited adolescents [23, 24, 33, 34]. Compared with the study conducted by Dumith and colleagues [24], who employed the same definitions for smoking, alcohol consumption and physical inactivity among 3990 adolescents who were 14–15 years old from the 1993 Pelotas birth cohort in a medium-sized city in Southern Brazil, the present findings demonstrated that Malaysian adolescents had a lower prevalence of alcohol use (5.0% in the present study vs 24.8%) but a higher prevalence of smoking (14.6% in the present study vs 5.7%) and higher rates of physical inactivity (85.5% in the present study vs 70.4%). Nonetheless, in contrast to Brazilian adolescents, the prevalence of smoking and alcohol use were lower among Malaysian girls than boys. The disparity in the prevalence of smoking and alcohol use by gender that has been reported across countries [35, 36] could possibly be attributable to the differences in social norms and cultural context. This is because the social norms of smoking and alcohol use among females are generally not acceptable by Malaysian societies, and therefore, these beliefs prevent girls from engaging in such risk behaviours [7, 37].

Regarding the prevalence of multiple lifestyle risk behaviours, the prevalence of simultaneous occurrences of at least 2 risk behaviours (80%) among Malaysian adolescents was higher than that reported among Brazilian adolescents [24], Canadian adolescents [34] and English and Brazilian adult populations [38]. On the other hand, the present findings were consistent with previous studies among Australian adolescents [33] as well as Dutch [16] and Swiss [39] adults such that a similar prevalence of multiple risk behaviours was observed in both genders. These findings suggest that intervention programmes using a multiple-behaviour approach instead of a siloed approach should be advocated among adolescents, regardless of gender, in view of the rather high prevalence of multiple lifestyle risk behaviours.

In contrast to previous works that investigated only the prevalence of co-occurring risk behaviours [23, 40] but not clustering effects, the present study demonstrated that significant clustering of lifestyle risk behaviours was observed in 16 and 15 out of 32 different combinations of the five lifestyle risk behaviours of concern in boys and girls, respectively. The clustering of multiple risk behaviours demonstrated in the present study substantiated the findings from previous studies involving both adolescents [24, 33, 34] and adults [25, 26, 41]. Furthermore, in concordance with previous literature, smoking and alcohol consumption were prone to cluster either alone or together with other risk behaviours in both genders [25, 34, 39, 41, 42]. The clustering of risk behaviours observed in the present study follows Jessor’s problem-behaviour theory that adolescents who are engaged in one problem behaviour tend to also be involved in other problem behaviours due to the shared linkages of such behaviours in the social ecology of adolescence [21]. This suggests that joint interventions on risk behaviours that have been demonstrated to be more efficient and effective [43] should be preferred over individual behaviour interventions in order to combat the synergistic effect of clustered risk behaviours on the well-being of adolescents.

In general, more profound clustering effects of lifestyle risk behaviours were observed in girls than in boys, and such findings corroborated previous findings [25, 26]. In the present study, although a higher observed prevalence (O, the numerator in O/E ratio) was found in most combinations of lifestyle risk behaviour among boys than among girls, the lower observed prevalence of individual risk behaviours in girls had given rise to a lower expected prevalence (E, the denominator in O/E ratio) for a particular combination of risk behaviours, which subsequently increased the O/E ratios. Therefore, more profound clustering effects of lifestyle risk behaviours were observed among the girls. In addition, another plausible reason is that although girls are generally known to have lower risk-taking behaviour (as shown by their lower prevalence of individual behaviours in the present study, except for physical inactivity), a study demonstrated that female adolescents who engaged in high-risk behaviour tend to be more involved in other high-risk behaviours than their male counterparts [44]. Furthermore, a higher level of perceived stress [45], tendency to ruminate and feelings of helplessness [46] among girls could predispose them to engage in dysfunctional coping measures such as risk behaviours to deal with stress [47]. Despite the observation of marked clustering of risk behaviours (higher O/E ratios) among girls in both the present study and previous literature, multivariable logistic analyses demonstrated that the likelihood of engaging in clusters of risk behaviours was independent of socio-demographic factors among girls. Previous studies have noted that adolescent girls were more likely to be influenced by psychosocial factors [45, 46] and therefore more likely to engage in unhealthy risk behaviours [47]. As such, in-depth investigations into the actual causal factors in future studies, particularly from the psychosocial perspective, could probably shed some light on the significant determinants of the clustering of lifestyle risk behaviours among Malaysian girls.

In contrast, being Malay or having at least 3 close friends was protective against engagement in clusters of risk behaviours in boys. As posited by social control theory [48], one of the plausible reasons for Malay (which is synonym to Muslim in Malaysia) boys being less prone to engage in clusters of lifestyle risk behaviours could be their strong proscriptive norm against consumption of alcoholic drinks [49], as compared to other ethnic counterparts in Malaysia. On the other hand, social relationships, which can be measured by the number of friends, are an important element in school connectedness [50]. School connectedness is an essential protective factor against a number of risk behaviours among adolescents because adolescents with good school connectedness believe that they are cared for by adults and peers as an individual [51] and are emotionally well [50], thereby reducing their likelihood of engaging in unhealthy behaviours. As such, the present findings corroborated the above speculation that boys with at least 3 close friends were less likely to engage in clusters of lifestyle risk behaviours than their counterparts who had fewer than 3 close friends. However, the negative association between the number of close friends and engagement in multiple risk behaviours should be further scrutinised since the health risk behaviours and other contextual factors of the respondents’ close friends were not investigated in the present study. In terms of family influence, the nonsignificant association of parents’ marital status but the significant association of the number of close friend with the clustering of risk behaviours provided some evidence supporting the theories of human development, which stipulated that adolescents are more influenced by their peers than by their parents because they have more common characteristics with their peers [52]. Another plausible reason for this nonsignificant association is that other important family-related determinants such as family cohesion [53] and parental conventionality [54] were not investigated in the present study and therefore should be explored in future studies.

The present study had a few limitations. First, the cross-sectional nature of the study precluded causal claims on the sequence of engagement in the five lifestyle risk behaviours. Second, the inclusion of school-going adolescents aged 16–17 years limited the generalisation of the present findings to adolescents of other age groups and those not enrolled in upper secondary school (the enrolment rate of upper secondary school was 84.75% in 2013) [55]. Third, the self-reported responses for all five risk behaviours might have given rise to under-reporting due to social desirability bias, especially for alcohol use. Fourth, the use of a binary outcome variable in the multivariable logistic regression could have limited the interpretation of the associations between sociodemographic factors and clustering of lifestyle risk behaviours. However, there were a few reasons for the dichotomisation of the outcome variable. First, we aimed to identify the sociodemographic determinants of those who were more likely to have clustered lifestyle risk behaviours, as compared to their counterparts who did not have clustered lifestyle risk behaviours, regardless of the type of combination, because there are too many combinations (32 combinations) to be scrutinised individually. Second, some combinations had fewer respondents (less than 20), and given the number of predictors in the regression model, dichotomising the outcome variable would be a better option for a valid and reliable results. Third, underscoring the efficiency of cluster analysis, we had run a two-step cluster analysis, and a total of 7 discernible clusters were deduced. A multinomial logistic regression of this 7-level outcome variable with the same set of predictors as the binary regression model was performed, however, we found that there were a total of 1028 (69.3%) cells (i.e. cluster levels by predictors) with zero frequencies and therefore made findings from the multinomial regression less reliable. Nonetheless, despite the limitations, the present study is of particular relevance and importance, as it is the first study to provide a holistic illustration of different combinations of clustered lifestyle risk behaviours and the magnitude of clustering rather than individual behaviours among school-going Malaysian adolescents.

## Conclusion

The present study clearly demonstrated the clustering of a number of specific combinations of lifestyle risk behaviours among Malaysian adolescents. Early intervention programmes that target the high-risk groups identified in the present study are greatly needed to reduce the increasing burden of non-communicable diseases in Malaysia, which are commonly attributable to the five major modifiable lifestyle risk behaviours studied.

## Data Availability

The datasets for the MyAHRB study are not publicly available due to government policies. Data are, however, available from the corresponding author (Teh CH) upon reasonable request and with the permission of the Director-General of Health, Malaysia.

## Abbreviations

aOR: Adjusted odds ratio
BMI: Body mass index
CI: Confidence interval
E: Expected prevalence
GSHS: Global School Health Survey
MREC: Medical Review and Ethics Committee
MyAHRB: Malaysian Adolescent Health Risk Behaviour
O: Observed prevalence
O/E: Observed prevalence-to-expected prevalence
